# Pregnancy-related acute kidney injury in the African continent: where do we stand? A systematic review

**DOI:** 10.1007/s40620-022-01349-2

**Published:** 2022-06-16

**Authors:** Ahmed Saad Shalaby, Rasha Samir Shemies

**Affiliations:** 1 Mansoura-Manchester Medical Program, Mansoura Faculty of Medicine, Mansoura University, Mansoura, Egypt; 2 Mansoura Nephrology and Dialysis Unit, Mansoura University, Mansoura, Egypt

**Keywords:** Acute kidney injury (AKI), Pregnancy, Pregnancy-related acute kidney injury (PR-AKI), Africa

## Abstract

**Background:**

Pregnancy-Related Acute kidney injury (PR-AKI) is a global health problem with substantial maternal and fetal morbidity and mortality. However, little is known about the current situation in the developing world including African countries. Africa is the poorest continent per capita, and women from Sub-Saharan Africa alone account for 66% of the estimated global maternal deaths from preventable obstetric causes.

**Methods:**

**Objective:**

To review the literature on the clinical profile, maternal and renal outcomes of women with PR-AKI in the African continent.

**Search strategy:**

Medline, ISI Web of Science, Scopus, and Cochrane library were searched in February 2022, using the MeSH terms and text key words: “pregnancy”, “pregnant”, “acute kidney injury”, “acute renal insufficiency”, “acute renal injury”, “acute renal failure”, and “Africa”.

**Selection criteria and data collection:**

Studies from African countries which reported maternal and renal outcomes in women with PR-AKI during pregnancy or postpartum were included. Editorials, short communications, and case reports were excluded. The study quality was assessed using the NHLBI tool. Data extraction was done using predefined data fields.

**Results:**

A total of 167 studies were evaluated, of which 14 studies from seven African countries met the inclusion criteria. Preeclampsia, obstetric hemorrhage, and sepsis represented the main causes of PR-AKI. Maternal mortality ranged between 0 and 34.4%. Although the majority of women needed ICU admission and hemodialysis, renal recovery occurred in 53.1–90% of patients. Perinatal mortality has been reported to be 1.5–60.5% in the included studies.

**Authors’ conclusions:**

PR-AKI in Africa represents the second leading cause of AKI. Limited access to obstetric care, late referral, and late diagnosis of women with risks for PR-AKI hinder the curtailment of the problem. Provision of health care facilities with adequately trained personnel and implementation of preventive strategies will be of great value in decreasing the magnitude of the problem.

## Background

**Fig. 1:**
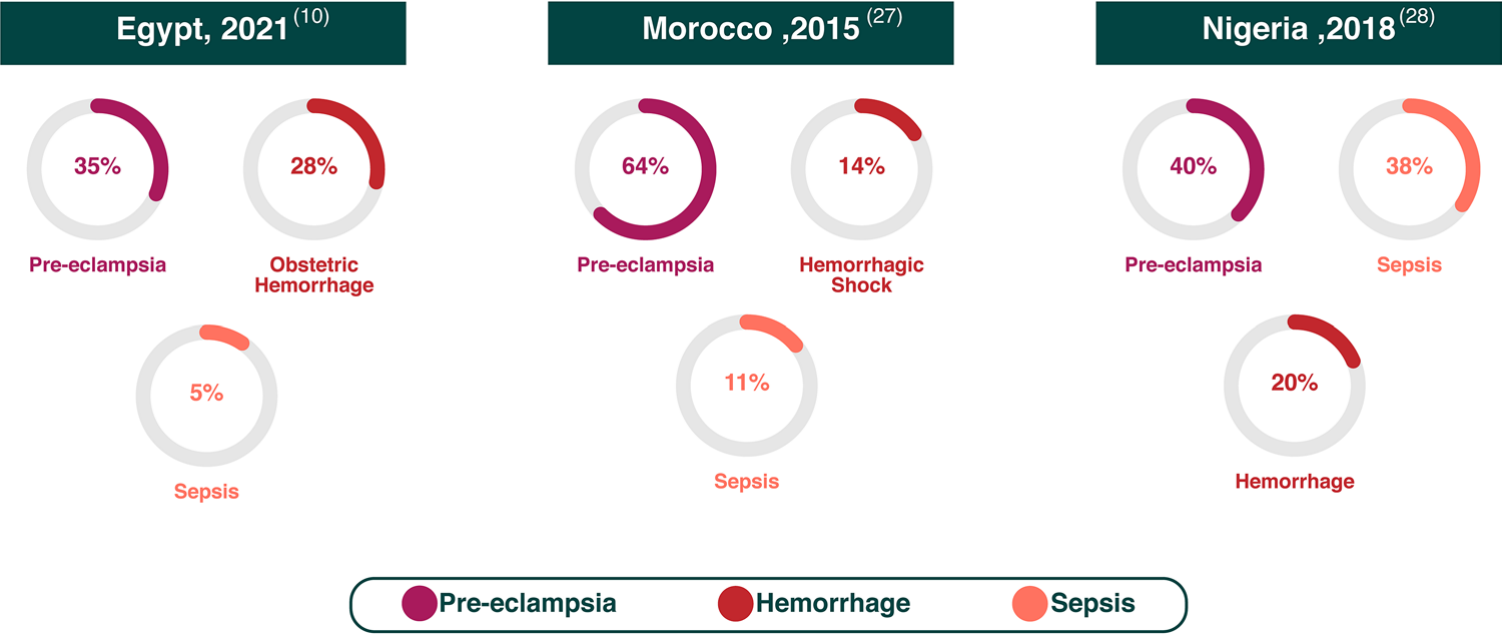
Main causes of PR-AKI in different African countries

Pregnancy-Related Acute kidney injury (PR-AKI) is a life-threatening complication of pregnancy, characterized by an abrupt decline in kidney function during pregnancy and puerperium. It represents a major public health problem with substantial maternal and fetal morbidity and mortality as well as a higher risk of progression to End-Stage Kidney disease [1–5]. It is thought that pregnancy increases the risk of AKI by 51% [6]. The significant improvement in obstetric healthcare and reduction of septic abortions in the past few decades might have contributed to globally decreasing the burden of the problem [7], however, little is known about the current situation in many developing countries including African countries [8–10]. In high-income countries, while PR-AKI is a rare entity, its incidence has recently increased [3, 11–15]. This is attributable to different risk profiles including advanced maternal age at conception, obesity, the use of assisted reproduction technology and increased incidence of pregnancy in women with chronic hypertension and chronic kidney disease (CKD) [1]. In low-income countries, lack of adequate perinatal care and inappropriate management of pregnancy-related complications still represent the leading causes of PR-AKI [5, 9]. A global, cross-sectional study, conducted by the International Society of Nephrology (ISN) to expand evidence about the epidemiology of AKI in low- and middle-income countries demonstrated that pregnancy is one of the most common causes of AKI in these countries [16]. The incidence of PR-AKI is much higher in developing countries (4–26%) when compared to developed countries (1.0–2.8%) [3, 17, 18]. Africa is the world's second-largest and second-most populous continent after Asia, with 1.3 billion people in 2018 [19, 20]. Africa is the poorest continent per capita, which may be attributed to the colonial activities by Western nations and deleterious domestic policies [21]. African women and girls account for the majority of all global deaths from preventable causes related to pregnancy and childbirth. Sub-Saharan Africa alone accounted for 66% of the estimated global maternal deaths in 2015 [22]. PR-AKI still represents a serious burden for the health systems across the African continent, however, the problem is neither adequately studied, reported, nor considered. In Africa, AKI related to obstetric causes came in second place among all causes of AKI. The incidence of PR-AKI in Africa is ~ 1 in 1,000 deliveries, accounting for 5–27% of all cases of AKI among adults in Africa, which is 20–100-fold higher than in developed countries [22–24]. A systematic review including 41 studies concerning AKI in Sub-Saharan Africa revealed that obstetric causes represent 16% of all AKI causes, with septic abortion as the leading cause [25]. Pre-eclampsia, obstetric hemorrhage and sepsis represent the main underlying causes of PR-AKI in African countries, with differences in their ranking as shown in Fig. 1 [26]. It has been pointed out that the limited access to obstetric care, late referral, and late diagnosis of women at risk for PR-AKI represent the main barriers that currently need to be dealt with [27–29]. Great strides need to be made in order to reduce the magnitude of the problem and save maternal and fetal lives, beginning with the identification and assessment of the risk factors and clinical patterns of the problem to assist in the decision-making process.

The scope of this review is to highlight the current literature concerning PR-AKI in Africa, reporting the clinical profile and outcomes of PR-AKI across African countries.

## Methods

### Search strategy

We conducted a systematic review of the literature in accordance with preferred reporting items for systematic reviews and meta-analysis guidelines (PRISMA) [30]. A Comprehensive literature search was conducted in PubMed, ISI Web of Science, Scopus, and Cochrane library, considering all eligible studies up to February 2022, using the MeSH terms and text key words: “pregnancy”, “pregnant”, “acute kidney injury”, “acute renal insufficiency”, “acute renal injury”, “acute renal failure”, and “Africa”. The protocol for this systematic review was registered on the International Prospective Register of Systematic Reviews (PROSPERO) under the registration number CRD42022322028.

### Inclusion and exclusion criteria

Original articles in English from any African country and reported maternal and renal outcomes in women with PR-AKI during pregnancy or postpartum were included and screened for eligibility. Articles in languages other than English, letters to the editor, editorials, short communications, and case reports were excluded, as were studies with no defined criteria for the diagnosis of PR-AKI. The titles and abstracts were screened independently by two investigators (AS and RS). Furthermore, references of previous review articles were also analyzed for any relevant citations. Any discrepancy between the two authors on study eligibility was resolved through discussion.

### Data extraction and outcomes

A.S and R.S independently extracted data regarding baseline characteristics from the included studies using a standardized Excel sheet. The following information was retrieved from each article: country of the study, total number of included patients, number of patients with PR-AKI and definition criteria of PR-AKI. Pregnancy outcomes were also extracted and were divided into maternal and fetal outcomes. Maternal outcomes included: maternal survival and need for intensive care unit (ICU) admission. Fetal outcomes included: premature delivery and fetal mortality (whether reported as stillbirth or perinatal mortality). Finally, renal outcomes were extracted and included: need for dialysis, renal recovery, non-recovery (progression to CKD or remaining dialysis-dependent).

### Quality assessment

The study quality was assessed using the National Heart, Lung, and Blood Institute's standardized Quality Assessment Tool for Observational Cohort and Cross-Sectional Studies (NHLBI tool) [31]. The NHLBI tool includes 14 questions regarding study methodology involving study population, sampling procedures, and sample size; exposure and outcome measurements; statistical analyses; and sources of confounding. The NHLBI tool aims to evaluate the internal validity of the individual studies and thus lacks a pre-specified scoring system. We used the same scoring system applied by Maass et. al. in their systematic review, where the quality of the included studies was categorized according to percentage score ranging from 0 to 100% into 4 categories: poor (0–25%), fair (26–50%), good (51–75%) or excellent (76–100%) [32]. Discrepancies in assessment of study characteristics were resolved as a group.

Due to heterogeneity of study populations, exposures and outcome measures reported, we did not apply quantitative analysis of the results (meta-analysis). The outcomes and characteristics of the study populations are simply reported as numbers and percentages.

## Results

**Fig. 2:**
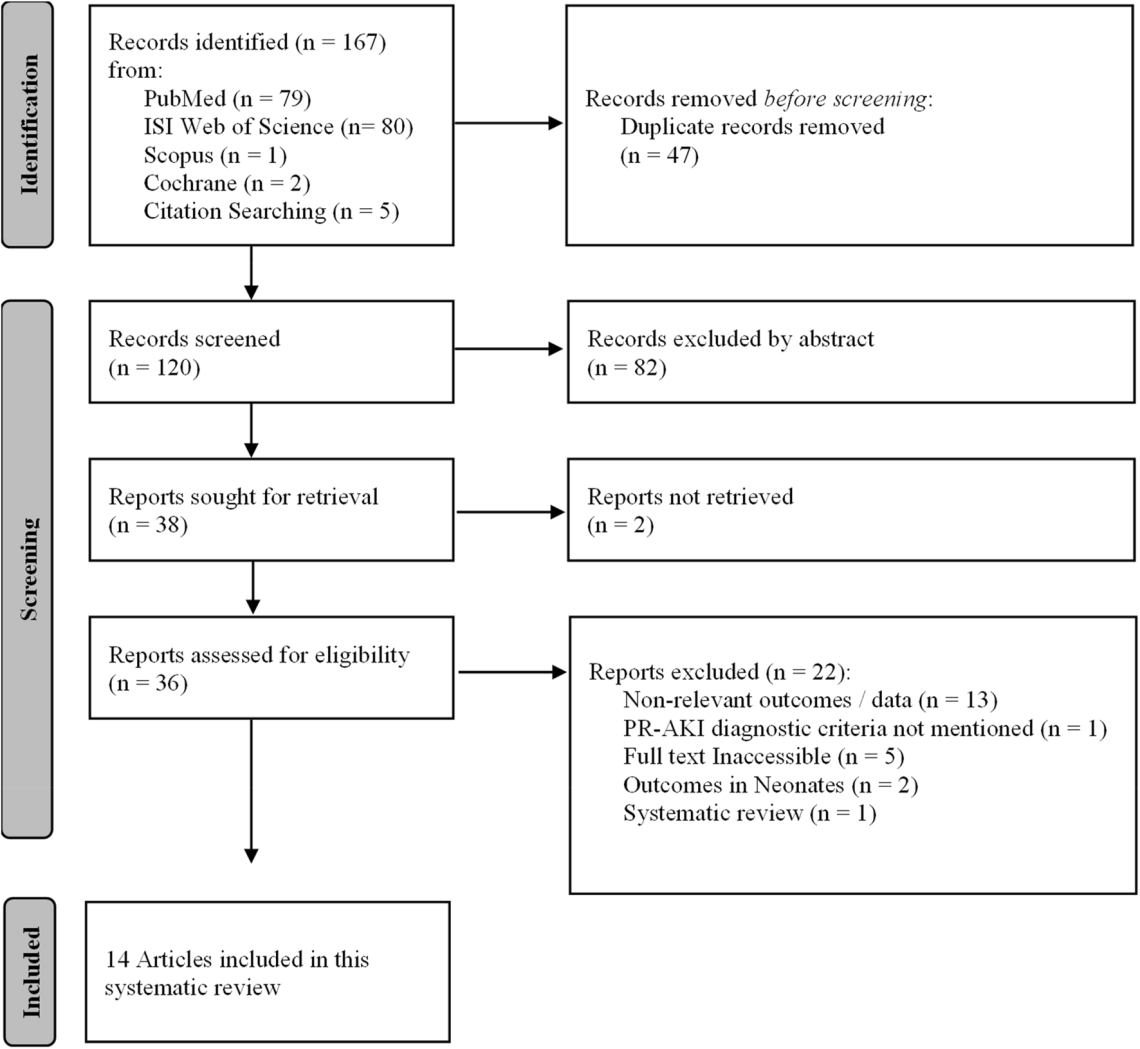
PRISMA flow chart of this systematic review

**Fig. 3:**
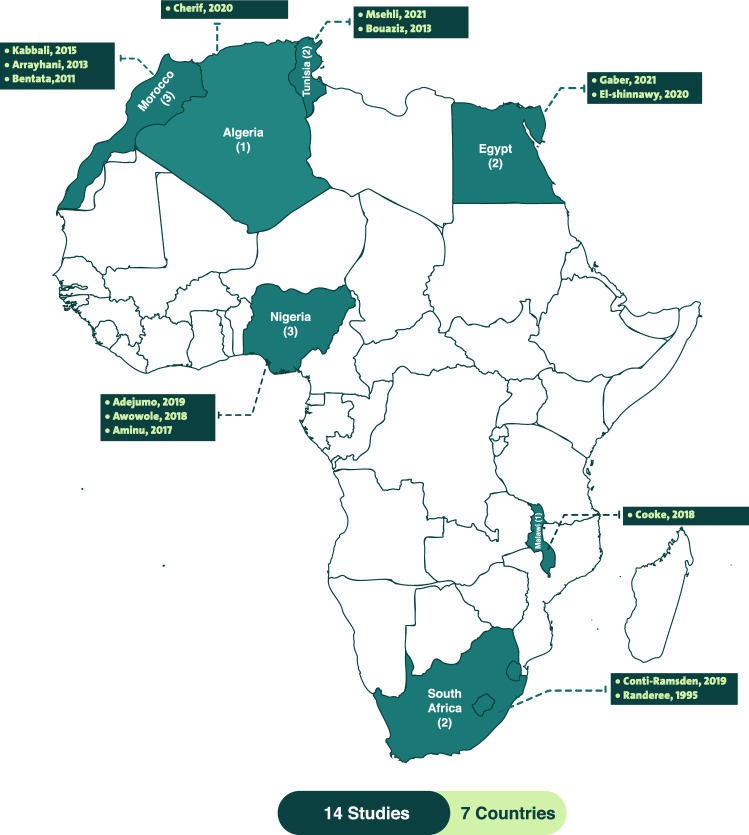
Distribution of the 14 studies across Africa

Literature search identified 167 studies in total. Fourteen studies met the inclusion criteria and include 1233 women with PR-AKI (Fig. 2). Table 1 summarizes the characteristics of the data extracted from the 14 studies. The studies were carried out between 1995 and 2021. All of the included studies were observational; seven of which were prospective studies and the remaining seven were conducted retrospectively. Of the 14 studies, two included only patients with PR-AKI who underwent dialysis, and two others included only patients who were admitted to the ICU because of PR-AKI. The studies were carried out in seven African countries (Fig. 3); eight were conducted in North Africa (two from Egypt, three from Morocco, two from Tunisia and one from Algeria), three studies were carried out in West Africa (Nigeria) in addition to two studies from South Africa and one form Malawi. PR-AKI was defined variably across the 14 studies; five studies defined it according to KDIGO criteria (Kidney Disease Improving Global Outcomes), five studies used the RIFLE (Risk, Injury, and Failure; and Loss, and End-stage kidney disease) criteria, one study defined PR-AKI using either RIFLE OR stage III AKIN OR KDIGO. The three remaining studies variably defined PR-AKI based on changes in urine output and/or serum creatinine. The NHLBI tool was used to assess the quality of the included studies. According to the scoring system adopted, eight studies were rated as fair quality, fives studies were rated as good quality, one study was evaluated as excellent quality, and no study was evaluated as poor. To reduce publication bias, we did not include case reports. Since most of the included studies were of fair quality and quite heterogeneous, and the meta-analyses were not sensitive enough, the presentation of the results is merely descriptive. Table 2 summarizes the quality rating of the individual studies.

**Table 1:** Characteristics of the included studies

Study	Country	Study design	Total Number of studied patients	Pregnancies with AKI (*n*)	Definition criteria of AKI	Maternal mortality	ICU admission	Full recovery rate	Need for dialysis	Non-recovery (developed CKD/dialysis-dependent)	Premature delivery	Fetal mortality (stillbirth/perinatal mortality)
Gaber, 2021[10]	Egypt	Prospective	4,500	40	KDIGO	22.5%	NM	62.5%	NM	37.5%	15.0%	45.0%^a^
El-shinnawy, 2020 [40]	Egypt	Prospective	13,050	78	KDIGO	14.0%	NM	60.0%	15.3%	7.6%	NM	NM
Conti-Ramsden, 2019 [39]	South Africa	Prospective	1,547	237	KDIGO	3.0%	48.52%	80.4%	1.7%	2.6%	NM	37.1% ^b^
Randeree, 1995 [41]	South Africa	Retrospective	263	42^c^	Rising serum urea in the presence of falling urine Output and/or fluid overload in the presence of oliguria (urine output < 400 mL/day)	4.8%	NM	90.5%	100.0%	NM	NM	54.8%^b^
Adejumo, 2019 [34]	Nigeria	Retrospective	32	32	RIFLE	34.4%	15.60%	53.1%	75.0%	3.1%	NM	50.0%^d^
Awowole, 2018 [28]	Nigeria	Retrospective	43	40^c^	RIFLE OR stage III AKIN OR KDIGO	17.5%	NM	72.5%	100.0%	NM	24.1%	34.4%^b^
Aminu, 2017 [35]	Nigeria	Retrospective	132	26	Sudden onset oliguria (urine output < 400 mL/day) or anuria (urine output < 100 mL/day) with serum creatinine 1.5 mg/dl (132.6 µmol/l)	30.8%	NM	65.4%	26.9%	3.8%	NM	NM
Cooke, 2018 [29]	Malawi	Prospective	322	26	KDIGO	0%	NM	84.6%	0%	NM	NM	1.5%^b^
Kabbali, 2015 [27]	Morocco	Prospective	44	44	RIFLE	11.4%	NM	66.0%	38.6%	NM	NM	15.9%^b^
Arrayhani, 2013 [36]	Morocco	Prospective	5600	37	RIFLE	NM	NM	76.0%	16.2%	5.4%	NM	NM
Bouaziz, 2013 [42]	Tunisia	Retrospective	550	313^e^	Serum creatinine level > 0.8 mg/dL	6.0%	100%	NM	1.7%	0.4%	NM	NM
Bentata, 2011 [37]	Morocco	Retrospective	43	43^e^	RIFLE	25.6%	100%	84.4%	11.6%	NM	NM	60.5%^b^
Cherif, 2020 [43]	Algeria	Prospective	538	179^e^	KDIGO	20.0%	NM	NM	18.9%	6.7%	NM	NM
Msehli, 2021 [38]	Tunisia	Retrospective	96	96^e^	RIFLE	13.0%	NM	75.0%	23.0%	4.0%	NM	NM
Total	14											

*NM* Not mentioned, *KDIGO* Kidney Disease Improving Global Outcomes, *RIFLE* Risk, Injury, and Failure; and Loss, and End-stage kidney disease

^a^Stillbirth

^b^Perinatal Mortality

^c^Patients with PR-AKI, who only underwent dialysis

^d^Simply described as Fetal mortality

^e^Patients with PR-AKI, who were only admitted to the ICU

**Table 2:** Quality assessment of individual studies using the NHLBI tool for quality assessment

Questions	Gaber, 2021[10]	El-shinnawy, 2020 [40]	Conti-Ramsden, 2019 [39]	Randeree, 1995 [41]	Adejumo, 2019 [34]	Awowole, 2018 [28]	Aminu, 2017 [35]	Cooke, 2018 [29]	Kabbali, 2015 [27]	Arrayhani, 2013 [36]	Bouaziz, 2013 [42]	Bentata, 2011 [37]	Cherif, 2020 [43]	Msehli, 2021 (38)
Was the research question or objective in this paper clearly stated?	Yes	Yes	Yes	Yes	Yes	Yes	Yes	Yes	Yes	Yes	Yes	Yes	Yes	Yes
Was the study population clearly specified and defined?	Yes	Yes	Yes	Yes	Yes	Yes	Yes	Yes	Yes	Yes	Yes	Yes	Yes	Yes
Was the participation rate of eligible persons at least 50%?	Yes	Yes	Yes	Yes	Yes	Yes	Yes	Yes	Yes	Yes	Yes	Yes	Yes	Yes
Were all the subjects selected or recruited from the same or similar populations (including the same time period)? Were inclusion and exclusion criteria for being in the study prespecified and applied uniformly to all participants?	Yes	Yes	Yes	Yes	Yes	Yes	Yes	Yes	Yes	Yes	Yes	Yes	Yes	Yes
Was a sample size justification, power description, or variance and effect estimates provided?	No	No	No	No	No	No	No	No	No	No	No	No	No	No
For the analyses in this paper, were the exposure(s) of interest measured prior to the outcome(s) being measured?	Yes	Yes	Yes	No	No	No	No	Yes	Yes	Yes	No	No	No	No
Was the timeframe sufficient so that one could reasonably expect to see an association between exposure and outcome if it existed?	Yes	CD	Yes	CD	CD	CD	CD	CD	CD	CD	CD	CD	CD	CD
For exposures that can vary in amount or level, did the study examine different levels of the exposure as related to the outcome (e.g., categories of exposure, or exposure measured as continuous variable)?	Yes	Yes	No	No	Yes	No	Yes	Yes	Yes	Yes	No	No	No	No
Were the exposure measures (independent variables) clearly defined, valid, reliable, and implemented consistently across all study participants?	Yes	Yes	Yes	No	Yes	Yes	No	Yes	Yes	Yes	Yes	Yes	Yes	Yes
Was the exposure(s) assessed more than once over time?	Yes	Yes	Yes	CD	CD	CD	CD	Yes	Yes	Yes	CD	CD	Yes	CD
Were the outcome measures (dependent variables) clearly defined, valid, reliable, and implemented consistently across all study participants?	Yes	Yes	Yes	Yes	Yes	Yes	Yes	Yes	Yes	Yes	Yes	Yes	Yes	Yes
Were the outcome assessors blinded to the exposure status of participants?	No	No	No	No	No	No	No	No	No	No	No	No	No	No
Was loss to follow-up after baseline 20% or less?	Yes	Yes	Yes	NA	NA	NA	NA	Yes	Yes	Yes	NA	NA	CD	NA
Were key potential confounding variables measured and adjusted statistically for their impact on the relationship between exposure(s) and outcome(s)?	No	No	No	No	No	No	No	No	No	No	No	No	No	No
Summary quality	Excellent	Good	Good	Fair	Fair	Fair	Fair	Good	Good	Good	Fair	Fair	Fair	Fair

*CD* cannot determine, *NA* not applicable

Maternal outcomes were reported in the 14 included studies. Maternal mortality ranged between 0 and 34.4% in 13 of the 14 included studies. The need for ICU admission was reported in four studies, two of which solely investigated PR-AKI patients who were admitted to the ICU, while the other two studies showed a need for ICU admission in 15.6% and 48.52% of women with PR-AKI. Only eight studies reported fetal outcomes. Fetal mortality rate was either reported as stillbirth (one study), perinatal mortality (six studies) or was simply described as fetal mortality (one study). Perinatal mortality ranged between 1.5 and 60.5%. Two studies reported premature deliveries. Renal outcomes were reported in 13 of the 14 included studies. Two studies included patients with PR-AKI who only underwent dialysis and therefore have a reported dialysis rate of 100%, while the highest reported rate of dialysis in the remaining studies was 75%. Full recovery occurred in 53.1–90% of patients. Nine studies reported the long-term renal outcome (progression to CKD or remaining dialysis dependent).

### Clinical profile and characteristics of PR-AKI in the included studies

PR-AKI represented a major health problem with ominous outcomes in the studied countries. In Nigeria, the most populous African country and one of the six countries that collectively contribute to more than half of the total maternal deaths worldwide [33], Adejumo et al. in their retrospective study reported maternal and fetal mortality rates of 34.4% and 50%, respectively, in women complicated by PR-AKI, with ICU admission, hypotension and impaired consciousness as the main reported associations [34]. In the aforementioned study, most cases presented in the post-partum period, with 75% of subjects requiring hemodialysis, however, 50% of patients fully recovered and only 3.1% became dialysis-dependent [34].

Pregnancy-related acute kidney injury requiring dialysis was further investigated in Nigeria by Awowole et al. who reported PR-AKI requiring dialysis in 43 patients, all of whom presented with oliguria [28]. Unfortunately, 15% presented late with pulmonary edema, and all the women had elevated serum creatinine concentrations. Hemodialysis was the only administered modality of dialysis, and the number of dialysis sessions ranged between two and thirteen, with a median of three sessions. The duration of hospital stay ranged from one to 133 days, with a mean of 33 days. The maternal and perinatal mortality rates were 17.5% and 34.4%, respectively [28]. This was comparable to the study conducted by Aminu et al. who reported 26 patients with PR-AKI over a period of three years, of whom 61.5% did not receive proper antenatal care. Seven (26.9%) underwent hemodialysis, with a range of three to six dialysis sessions per patient. Maternal mortality of 30.8% was reported. Severe hemorrhage (15.4%), pulmonary edema (3.9%) and disseminated intravascular coagulation (DIC) complicating puerperal sepsis (3.9%) were the main causes of maternal deaths [35]. It appears that PR-AKI in Nigeria is largely caused by obstetric hemorrhage occurring in areas with poor access to health care and antenatal services, therefore a provision of health care facilities in high-burden areas is strongly needed.

In Egypt, the third most populous African country, PR-AKI accounted for 14% of all patients who presented to the renal service and 1% of women who presented to the obstetric service in a tertiary care hospital. Hypertensive disorders of pregnancy as well as obstetric hemorrhage were the key leading causes of PR-AKI. In this cohort, multiorgan failure complicated 27.5% of the included women; almost all presented to the hospital in severely compromised clinical conditions. Maternal and perinatal mortality rates of 22.5% and 45%, respectively, were reported [10].

On an initiative of the Moroccan Society of Nephrology, Kabbali et al. conducted a prospective national study to investigate the magnitude of PR-AKI in Morocco over six months. The study included 44 women, of whom 70.6% were illiterate and 66% did not receive antenatal care. Preeclampsia, followed by sepsis represented the two leading causes of PR-AKI in this cohort. Hemodialysis was required in 38.6% of cases. Five maternal deaths (11.4%) were reported: four due to septic shock and one to severe obstetric hemorrhage. Maternal age above 38 years and sepsis were identified as the main risk factors [27]. In an earlier Moroccan study, Arrayhani et al. similarly identified preeclampsia as the main cause of PR-AKI (66.6%), with 76% of the included women completely recovering kidney function [36]. Bentata et al. focused on the clinical features of women with PR-AKI exceeding 28 weeks of gestation on admission to ICU. Most patients in the study (51.2%) presented during the risk stage of RIFLE criteria, with 75% attributed to hypertensive disorders and hemorrhage. Thirty-nine point five percent of patients improved and were transferred to the maternity department 72 h after ICU admission, while 60.5% developed serious complications and required longer stays in the ICU. At three weeks post-ICU admission, 84.4% of patients had achieved full recovery, while 15.6% achieved partial recovery [37].

The Tunisian Bouaziz et al. conducted a retrospective data analysis of 550 women with PR-AKI over a period of 17 years in a Tunisian intensive care unit. Preeclampsia (66.5%) and acute hemorrhage (27.8%) constituted the main primary causes of PR-AKI. Serious complications were reported: retro-placental hematoma in 88 patients (16%), immediate postpartum hemorrhage in 59 patients (10.7%) and uterine rupture in 18 patients (3.3%). The study reported an overall maternal mortality of 6% which correlated to the severity of PR-AKI. Only 1.7% of women required dialysis and only two patients (0.4%) developed chronic renal failure and needed long-term dialysis, which is significantly lower than the rates reported in another Tunisian study by Msehli et al.; 23% and 4%, respectively [38]. In the cohort studied by Msehli et al., 16% of women diagnosed with PR-AKI required hospitalization and it was observed that most patients had received insufficient prenatal care (57%) [38].

Cherif conducted an observational, prospective study in two centers in East Algeria. He observed that among the 538 patients admitted to the ICU only during the postpartum period, 33.28% had AKI, and preeclampsia was identified as the most common cause (72.3%) [43].

In South Africa, a large prospective study of women admitted with preeclampsia identified 237 women with a diagnosis of PR-AKI based on the KDIGO criteria, representing 15% of admissions with preeclampsia. Past history of a hypertensive disorder of pregnancy represented the most substantial risk factor for the development of AKI and worsening AKI severity. Women who developed AKI, regardless of AKI stage, were more likely to die (risk ratio [RR], 4.3; *P* = 0.003), and to have a stroke (RR, 16.6; *P* = 0.015). In addition, women with PR-AKI were more likely to have a stillbirth (RR, 2.2; *P* < 0.001). Stillbirth rates significantly increased with increasing AKI severity. Seven maternal deaths (3.0%) were reported in women with PR-AKI. The overall perinatal mortality in offspring born to women with AKI was 37.1%. Fortunately, two-thirds of women had recovered from AKI at discharge [39].

Data from Malawi, one of the poorest countries in the world, revealed that the incidence of AKI among high-risk obstetric admissions is 8.1%. Preeclampsia/eclampsia (73.1%), antepartum hemorrhage (11.5%), and sepsis (11.5%) represented the main primary causes of AKI in this cohort. However, with tertiary nephrological and obstetric care, no patients with PR-AKI died or required dialysis and complete renal recovery was observed in 84.6% of patients. The perinatal mortality rate across all high-risk admissions was 13.8% [29]. The Malawi study concluded that maternal–fetal outcomes in Sub-Saharan Africa could be improved with earlier detection of hypertensive disorders in pregnancy.

## Discussion

The incidence, characteristics, and outcomes of AKI in general across the African continent are under-reported [24, 44, 45]. The current review was conducted to highlight the clinical profile and outcomes of PR-AKI in Africa. Across the 54 African countries, only 14 studies were accessible and met the eligibility criteria. It is noteworthy that there is no consensus definition or specific diagnostic criteria for PR-AKI in the literature, and different studies depend on different criteria that are used to diagnose AKI in the general population. Therefore, early detection of PR-AKI could be difficult with the use of the diagnostic criteria for AKI in the non-pregnant population [46, 47]. The various and wide physiological adaptations that occur in the renal and hemodynamic systems during pregnancy would explain the difficulty in establishing precise exclusive diagnostic criteria for AKI during pregnancy [13]. The different adopted diagnostic criteria led to discrepancies in the reported incidence and prevalence of conditions among various studies [42]. The majority of studies included in the review reported that prevalence and incidence of PR-AKI is still high in African countries, despite being a rare health problem in developed countries [10, 29, 48].

**Fig. 4:**
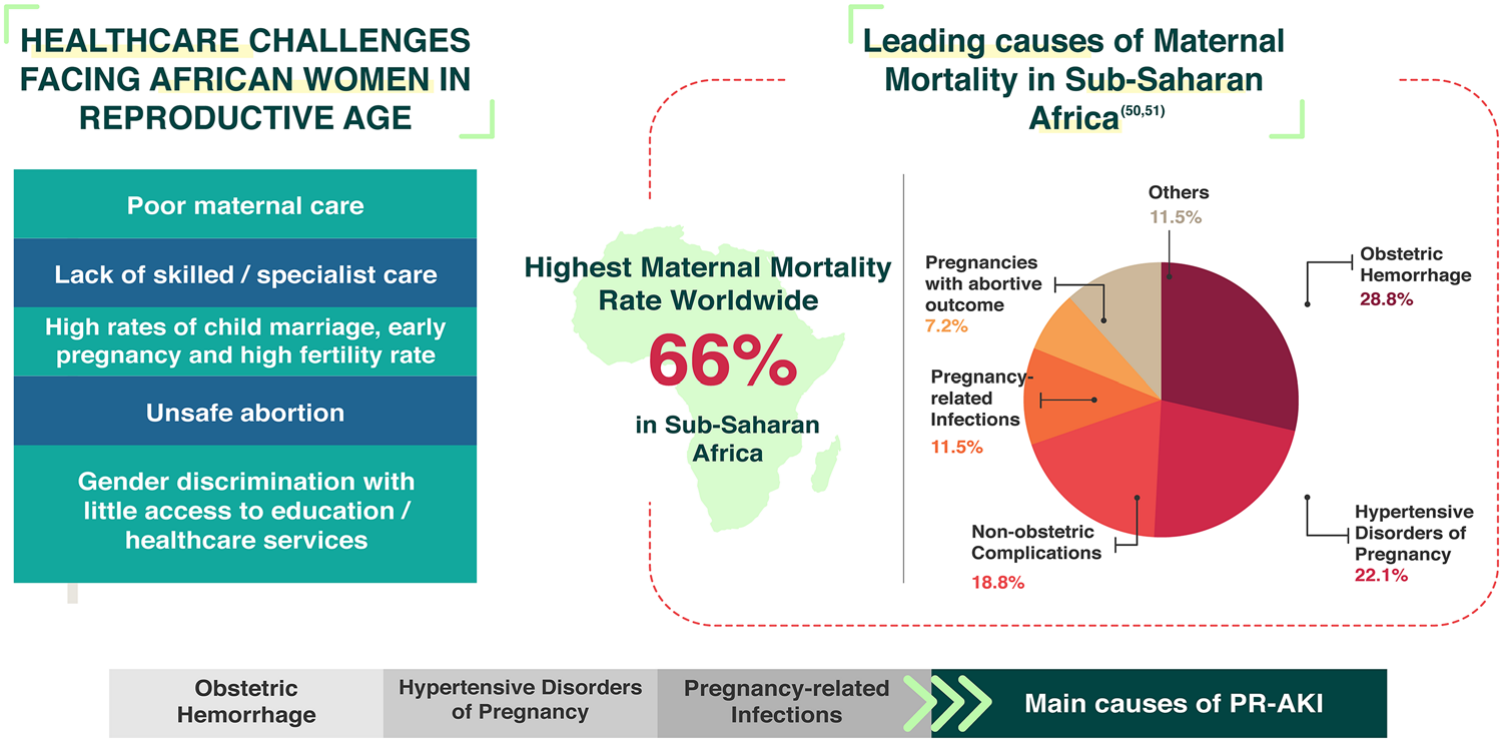
Healthcare challenges facing African women in reproductive age

Sub-Saharan Africa has the highest maternal mortality rate (66%) as reported by the Maternal Mortality Estimation Interagency Group (MMEIG) [49, 50]. The situation in African countries is devastating since maternal mortality is mostly due to preventable causes. The WHO Report of the Commission on Women’s Health in the African Region- 2012 stated clearly that: “Women in Africa bear a disproportionately large share of the global burden of disease and death, particularly in maternal morbidity and mortality” and enumerated various health challenges for African women in reproductive age, including: high fertility rate, poor maternal healthcare, lack of skilled care, high prevalence of AIDS/HIV, unsafe abortions and high maternal mortality rates (Fig. 4) [51].

The leading causes of maternal mortality in sub-Saharan Africa are obstetric hemorrhage (28.8%) followed by pregnancy-related hypertensive disorders (22.1%), and in fourth place comes pregnancy-related infections (11.5%), all of which are established risk factors for PR-AKI [5, 9, 50]. Acute Kidney Injury occurring during pregnancy represents a challenging clinical situation for health care providers [36]. It has been shown that PR-AKI increases the risk of perinatal mortality by 3.4-fold, and that it also increases the risk of pre-term deliveries and neonatal deaths [4, 5]. Maternal mortality and fetal loss have risen to 30–60% according to some studies [3, 18, 52]. Even after recovery, PR-AKI presents a long-term risk of pre-eclampsia and cardiovascular diseases including strokes. The adverse fetal outcomes such as preterm and small for gestational age deliveries in subsequent pregnancies have been additionally reported [53]. The risk of maternal mortality from pregnancy-related complications is 1:20 in Africa compared to 1:2000 in developed countries with hemorrhage, eclampsia and septic abortion constituting the main risk factors [8]. The incidence of PR-AKI, accordingly, might reflect the level of the obstetric care in high burden countries [9].

The most common presenting symptom for PR-AKI was oliguria/anuria [10, 28, 34, 38]. A shared feature among women in African countries is the poor and inadequate antenatal care in addition to late referral of cases to specialized tertiary centers [10, 27, 28, 34, 38]. Kabbali et. al reported that 66% of their Moroccan cohort lacked antenatal care [27]. Similarly, Msehli et. al. described that 57% of the Tunisian women included in their study had received insufficient prenatal care [38]. Additionally, 57.1% of the Nigerian deaths reported by Awowole et al. were attributed to the late referral of cases [28]. Hypertensive disorders of pregnancy (especially pre-eclampsia), obstetric hemorrhage and sepsis were the most common causes of PR-AKI in the included studies. Pre-eclampsia was the leading cause in nine studies [27–29, 36, 38–40, 42, 43], while obstetric hemorrhage was the leading cause in only two studies [10, 34]. In the majority of studies, sepsis was the second most widespread cause of PR-AKI, either after pre-eclampsia or obstetric hemorrhage. Carrying the risk of severe bleeding that predisposes to AKI, Cesarean Section (CS) was associated with higher incidence of PR-AKI in some studies [10, 28, 34]. Adejumo et al. suggested that this association could be improved by adequate resuscitation prior to CS as well as close pre- and post-operative monitoring of clinical signs of shock [34]. Additionally, Gaber et al. pointed out that anemia among women in developing countries impedes their ability to tolerate sepsis or obstetric hemorrhage [10]. Factors associated with increased risk of dialysis and ICU admission are HELLP syndrome (Hemolysis, Elevated Liver enzymes and Low Platelets), DIC, and placental abruption [38, 42]. Shock, DIC and prolonged ICU stay were the main causes associated with maternal death [10, 34]. All the included studies reported high maternal mortality rates in women with PR-AKI, with the exception of the Malawian study, which reported 0% mortality. This was thought to reflect the high quality of maternal care provided, early detection of the condition and multidisciplinary team management [29]. In general terms, the ominous outcomes attributed to PR-AKI could be explained in terms of low socioeconomic standards with regard to poverty, limited access to early and regular obstetric care, low levels of awareness and high fertility rate [18].

Around 1.5–2.5% of patients with PR-AKI progress to end stage renal disease and 4–9% of women with severe PR-AKI remain dialysis-dependent for 4–6 months post-partum and may progress to end stage kidney disease [5, 36, 54–57]. The risk of mortality increases when dialysis is required [58]. This progression to CKD adds an additional load onto the substantial health care burden in African countries, where overall prevalence of CKD in sub-Saharan Africa is reported to be 13.9% [59]. In an analysis conducted to measure the burden of CKD globally between 1990 and 2017, most African countries were found to have age-standardized rates between 500 and 999 disability-adjusted life-years (DALYs) per 100,000 population and the burden of CKD in sub-Saharan Africa was higher than expected when compared to the level of development [60]. Additionally, there is an expected rise in the number of patients receiving renal replacement therapy in Africa from 0.083 million in 2010 to 0.236 million by 2030 [61]. Nevertheless, data concerning the burden and etiology of CKD in African countries are still lacking and are unclear due to the lack of renal records in many African countries [62]. The risk of mortality increases when dialysis is required [58].

Accordingly, it seems apparent that obstetric ailments represent a major cause of AKI and mortality in Africa. PR-AKI still represents a continuous burden with menacing outcomes; however, it is largely underreported and neglected in almost all African countries.

Strenuous efforts and diligent work are needed in order to deal with the issue of PR-AKI in Africa. Different recommendations regarding management and prevention of PR-AKI have been published, however, they are insufficient and have been described as weak and having low grade quality of evidence [63]. Therefore, evidence of higher quality and extended data are needed to fill that gaps and to make improvements Multicenter controlled studies with expanded sample sizes should be adopted to bridge the present gaps in the current literature concerning PR-AKI [5]. Furthermore, researchers should provide follow-up data for patients with PR-AKI who undergo dialysis to demonstrate the rate and risk factors of progression to CKD and end-stage kidney disease. Recently, a few studies have suggested that various biomarkers could predict PR-AKI in early stages [47] and accordingly more effort should be made to investigate such biomarkers.

**Fig. 5:**
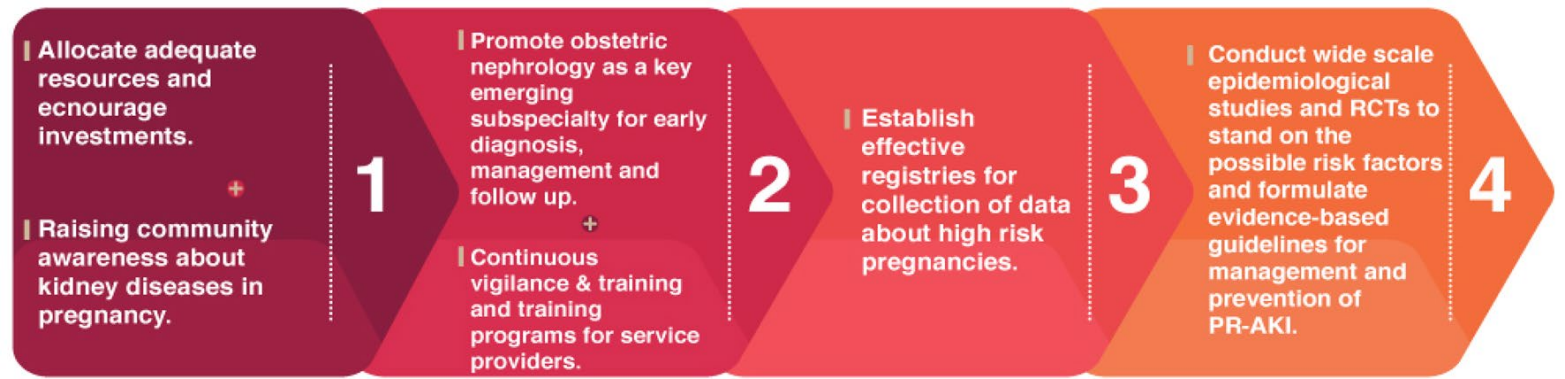
A suggested approach in an effort to improve the situation in the African setting

Raising community awareness about kidney diseases during pregnancy, the merits of their early diagnosis and the possible ominous outcomes is a cornerstone in tackling the issue. Additionally, specialized multi-disciplinary obstetric nephrology clinics would be of great value for providing early diagnosis of cases of PR-AKI as well as delivering proper short- and long-term management and follow up plans, not only for mothers with PR-AKI but also for their children that are born prematurely or small for gestational age [9, 26, 64]. Over the past few years, changes in the patterns of the etiology and characteristics of patients with PR-AKI have been observed [64], and these necessitate the establishment of effective updated registries that are involved in the collection and registration of data of high-risk pregnancies and their outcomes in order to identify the modifiable risk factors of PR-AKI and prevent them. Judicious use and application of proper lines of management of high-risk pregnancies require high levels of education, well-trained personnel and availability of resources which are lacking in many low-resource settings and should be promptly considered [65]. Consequently, allocation of adequate resources and investments in the prenatal care sector is mandatory in order to aid health care services and professionals in the early detection and proper management of high-risk pregnancies (pregnancies with obstetric hemorrhage, eclampsia, HELLP syndrome and sepsis). Figure 5 summarizes the approach we suggest in an effort to improve the situation in the African setting. The current systematic review was limited by the heterogeneity of data and studies about PR-AKI in African countries which prevented us from adding meta-analysis to the review. Moreover, small sample sizes and the lack of control groups in the included studies was another limitation. The low socioeconomic status and subsequent limited access to antenatal services challenges the curtailment of this deleterious trend. The significant maternal and fetal complications attributed to PR-AKI, especially in African countries, require healthcare authorities and professionals to pay serious attention to the matter. Provision of health care facilities with adequately trained personnel will be of value in decreasing the magnitude of the problem. International initiatives and strategies should be implemented to report and manage PR-AKI in high-burden communities. Table 3 enumerates a number of our recommendations that could be implemented in order to improve the outcomes of PR-AKI.

**Table 3:** Recommendations to prevent and decrease the magnitude of PR-AKI

1. Promote obstetric nephrology as a key emerging subspeciality through raising awareness and understanding
2. Introduce multi-disciplinary obstetric-nephrology clinics in tertiary centers for counseling and follow up of PR-AKI patients
3. Establish effective registries that are involved in the collection and registration of data of high-risk pregnancies and their outcomes
4. Upgrade the practice of obstetric nephrology through vigilance and training programs and establishment of obstetric-nephrology services in high-burden countries
5. Conduct multicenter wide-scale epidemiological studies with large sample sizes to identify and determine the actual burden of PR-AKI and the underlying risk factors in each nation
6. Implement national programs and aggressive public health initiatives aimed at screening and early identification of risk factors associated with PR-AKI
7. Raise awareness and empower pregnant women who experience kidney disease to get the best management and support at the right time
8. Advocate and lend a helping hand according to the Sustainable Development Goals (SDGs) pertaining to women’s health in Africa
9. Provide early referral of at-risk cases to specialized tertiary centers where multi-disciplinary teams are on call for control and follow-up of those cases
10. Allocate resources and investments to improve the prenatal care facilities and infrastructures as well as emergency obstetric services

## Conclusion

PR-AKI in Africa is a major public health problem contributing to maternal and fetal morbidity and mortality across the continent, however, the problem is neither adequately studied, reported, nor considered. More studies of high quality are needed to identify the real magnitude of the issue and plan out a management strategy.
