# Correction to: Pregnancy-related acute kidney injury in the African continent: where do we stand? A systematic review

**DOI:** 10.1007/s40620-022-01411-z

**Published:** 2022-07-25

**Authors:** Ahmed Saad Shalaby, Rasha Samir Shemies

**Affiliations:** 1 Mansoura-Manchester Medical Program, Mansoura Faculty of Medicine, Mansoura University, Mansoura, Egypt; 2 Mansoura Nephrology and Dialysis Unit, Mansoura University, Mansoura, Egypt

## Correction to: Journal of Nephrology 10.1007/s40620-022-01349-2

After publication online the author found that the reference citations in the text and reference list were incorrect.

The original article has been corrected.

